# Association between ultrasound morphologic features and histopathological findings of lobular carcinoma

**DOI:** 10.1002/jmrs.336

**Published:** 2019-08-31

**Authors:** Kathryn Malherbe, Philippa Bresser

**Affiliations:** ^1^ Department of Health Sciences, Department Radiographic Sciences University of Pretoria Pretoria South Africa

**Keywords:** Atypical lobular hyperplasia, histology, lobular carcinoma, morphology, predictors, ultrasound

## Abstract

**Introduction:**

Despite the incidence and recurrence rates of breast cancer, there are currently no biomarkers to predict which cases will develop into lobular carcinoma (LC).

The purpose of this study was to determine the association between ultrasound morphologic characteristics of LC and histopathological classifications.

**Methods:**

A retrospective, cross‐sectional study was conducted on the ultrasound images and histopathological reports of 100 patients with a confirmed LC diagnosis between January 2013 and December 2016.

**Results:**

Morphologic ultrasound characteristics most frequently reported in the dataset of positively diagnosed LC patients were; irregular ultrasound shape (86%), hypoechoic echogenicity (88%), poorly circumscribed margin (95%), posterior acoustic enhancement (93%) and absent calcifications (81%). Using Fisher's extract test, it was found that stromal fibrosis, single file type pattern, atypical lobular hyperplasia and LC Grade II were significantly correlated with irregular shape and hypoechoic echogenicity.

**Conclusion:**

A prognostic predictor tool can be designed from this study's findings which can then be used in practice to raise awareness of the unique morphometric markers related to LC of the breast.

## Introduction

Lobular carcinoma (LC) is the second most common carcinoma of the breast (5–20% incidence) and has a high risk of recurrence in the contralateral breast (50–70%).^1^, ^2^

Despite the incidence and recurrence rates of breast cancer, there are currently no biomarkers to predict which cases will develop into LC.^3^ Medical practitioners involved in the diagnosis, treatment and management of breast cancer rely on imaging reports and histopathologic confirmation of abnormal findings on mammograms and ultrasound.

Current research hallmarks mammography as the gold standard for breast cancer detection with a sensitivity rate of 85%. However, sensitivity is reduced to 68% with dense type breast tissue.^4^, ^5^

From a pathological and histological point of view, strict morphological criteria for LC and its various subtypes are uncommon.^6^ For example, infiltrating LC has marked reduction in diagnostic sensitivity due to malignant cells invading the surrounding stroma in rows and singular columnar cells; disrupting the underlying soft tissue structures. Microcalcifications are only seen with 1–2% of all diagnosed LC cases. Tumour cells characteristically surrounding the ducts without obstruction. This lack of ductal invasion is postulated to be associated with the lack of micro calcifications being present.^4^

The question arises whether there are certain morphometric properties of LC which are significantly correlated with positively diagnosed LC. Also, could the development of a prognostic predictor tool using such morphometric properties improve diagnosis of LC?

LC has a diffuse growth pattern, which relates to its low detection rate with mammography.^7^, ^8^ Infiltration of the breast tissue is diffuse and in a single row pattern of malignant cells, causing no destruction of the underlying normal breast tissue or reactive connective tissue. Thus, early stage and even late stage LC is rarely detected on mammograms.^4^

Ultrasound plays an integral role in the improved sensitivity and diagnosis of LC.^9^ It is commonly seen as a hypoechoic, irregular mass with indistinct margins in 85% of cases.^10^, ^11^, ^12^, ^13^

**Figure 1:**
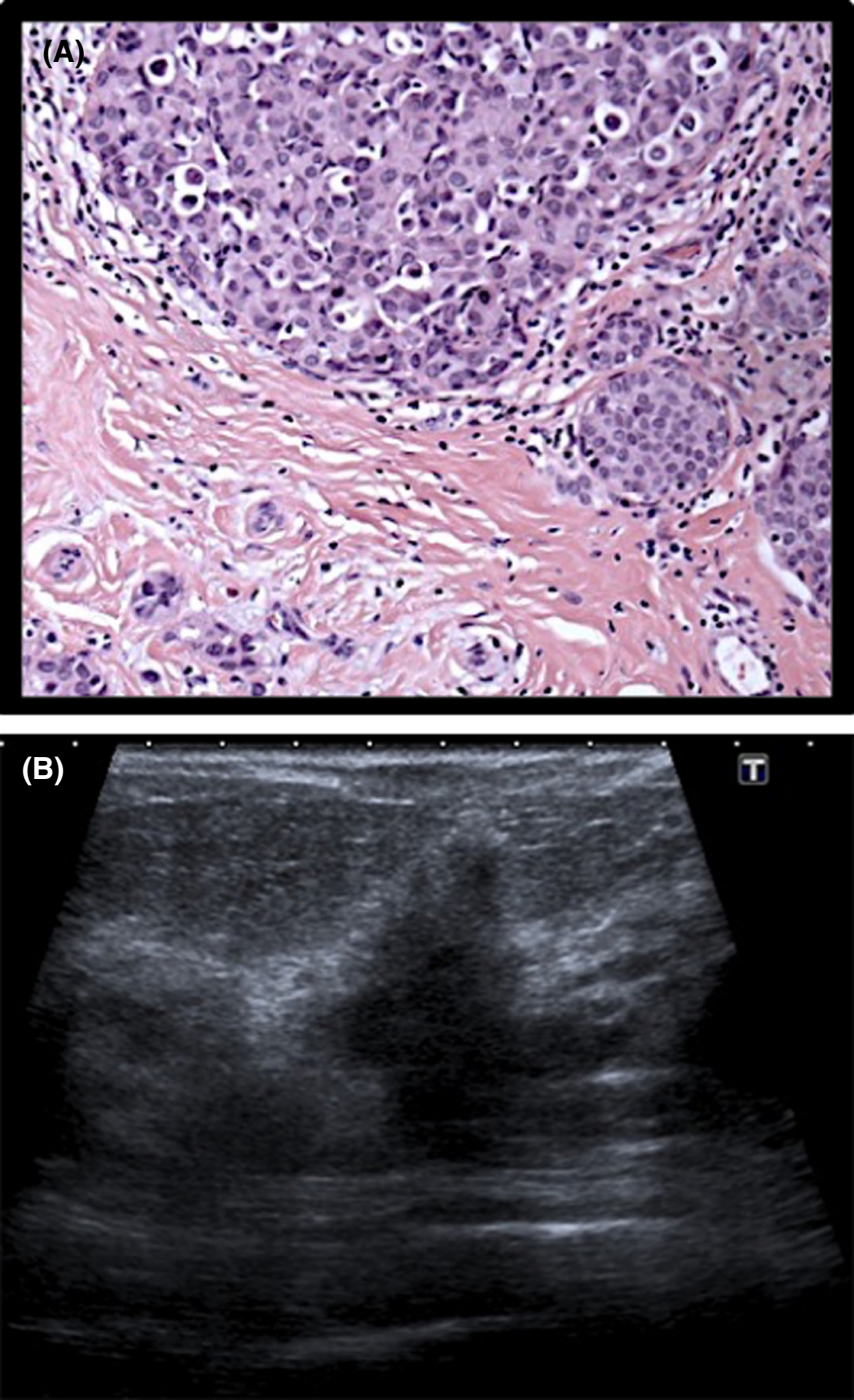
Histological and sonographic appearance of invasive lobular carcinoma. (A) Histology specimen of LCIS. (B) Ultrasound image of LCIS (poorly circumscribed mass).

Ultrasound findings of LC vary. Terms such as weak internal echogenicity, a heterogeneous (complex), hypoechoic mass, an irregular distribution or an angular, ill‐defined mass are used.^10^, ^11^ Irregular central shadow has been reported with pleomorphic type LC. Focal shadowing with no discrete mass present was reported with classic type LC (see Fig. 1). A lobulated well‐circumscribed mass was also mentioned in literature, associated with signet, alveolar and solid LC subtypes.^14^

It has been established that high‐frequency ultrasound is the preferred imaging modality for diagnosis of LC since mammography has limited sensitivity and specificity.^10^, ^15^ Varied previous literature are reported regarding morphometric properties of LC on ultrasound. Although the morphometric properties have been positively correlated with histopathologic results,^16^ there are variations in reported ultrasound morphometric characteristics of LC.^2^, ^16^, ^17^ Currently there is no instrument that can be used to assist in the specific diagnosis of LC from morphometric analysis of the ultrasound images.

## Material and Methods

A cross‐sectional descriptive study was conducted at two private radiology practices which both serve as dedicated women's wellness centres. Two radiologists are responsible for performing and interpreting the high‐frequency ultrasound examinations of the breast on the Siemens Acuson X300 5–13 MHz (Siemens, Germany) and the Toshiba Aplio 300 5–14 MHz linear array probes (Tecmed, South Africa). Data were collected retrospectively from patient files and prospectively from re‐observation of ultrasound images. Ethical clearance for use of retrospective ultrasound images was sought from Medical Research Ethics Committee of University of Pretoria.

Both radiologists have extensive knowledge regarding breast ultrasound of over 25 years.

The researcher handled the data securely and confidentially. Patient confidentiality was upheld through anonymity of personal information. No identifying information was used or reported on. Patient names were replaced with unique study numbers on the high‐frequency ultrasound images presented to the interpreters.

The Research Ethics Committee of the Faculty of Health Sciences waived the necessity of individual patient consent. However, consent was obtained from the radiology practices for access to the data which was essential for completion of the study.

### Measurement methods/techniques

Ultrasound images were retrieved from a local PACS system and were analysed on a five megapixel Barco workstation by two interpreters. All images were interpreted according to the guidelines of the BI‐RADS® lexicon. Interpreters completed the relevant section of the data collection sheet according to the findings on the high‐frequency ultrasound images. Interpreters were blinded to the histology results, ultrasound BIRADS classification and previous ultrasound reports.

Histopathological findings from retrospective results were all descriptions from specimens with terminology: atypical lobular hyperplasia, comedo necrosis, cystic dilated terminal lobular units, stromal fibrosis, epithelial hyperplasia, signet ring appearance, classic/single file/ single columns of linear cells or ribbons.

Once the interpreters had completed the relevant section of the data collection sheet, the researcher completed the remaining sections. Findings from high‐frequency ultrasound images were correlated with histology findings and ultrasound BIRADS classification to determine which morphometric characteristics were most accurate predictors of LC.

### Case selection

Consecutive sampling of all cases of histologically positive malignancies in the breast, diagnosed through ultrasound‐guided core biopsies from December 2016 retrospectively to January 2013 was undertaken. One hundred of the 1052 cases of patients with confirmed breast malignancy were confirmed LC cases. The ultrasound images of the confirmed LC cases were retrieved from the image database. The histology reports for each case were then extracted from the radiology database.

### Ultrasound image evaluation

Two radiologists evaluated the ultrasound images in terms of morphological characteristics which corresponded to the lesions on the high‐frequency ultrasound images for each case. Benign and malignant morphologic characteristics provided in the data collection sheet were derived from previous studies.^1^, ^17^, ^18^, ^19^, ^20^ Interpreters analysed all 100 cases independently and were blinded to the patient age, histological findings and ultrasound morphologic description of the retrospective radiology reports. Patient identifiers on the ultrasound images and histology reports were removed and allocated a unique study number prior to evaluation by interpreters.

### Histopathological reports

**Figure 2:**
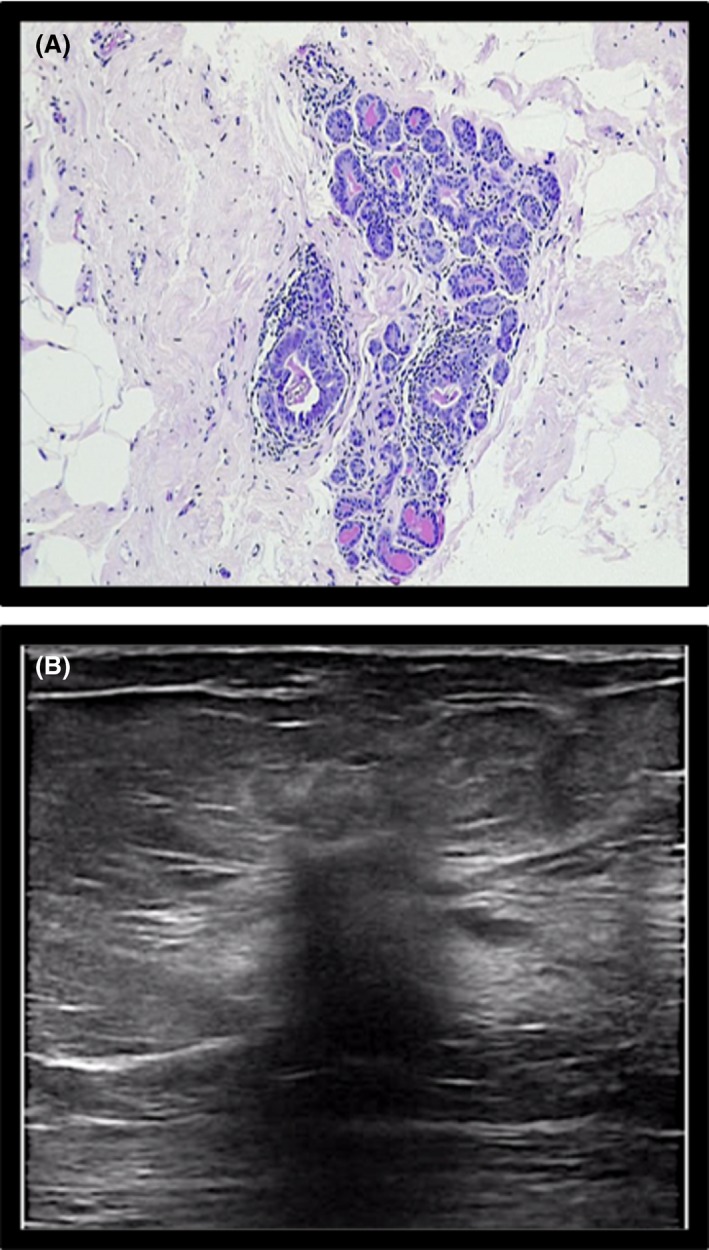
Histological and sonographic appearance of sclerosing adenosis/ epithelial hyperplasia. (A) Histology: epithelial hyperplasia, sclerosing adenosis. (B) Ultrasound: sclerosing adenosis. Note: Although benign finding, it is known to be in close proximity to early atypical hyperplasia in the breast tissue.

Histologic descriptions were grouped into five subsets; epithelial hyperplasia and stromal fibrosis (see Fig. 2), signet ring appearance, single file/ribbons/single file, comedo necrosis and cystic dilated terminal lobular units. Histologic grading was divided into four subsets namely LC Grade I, LC Grade II, LC Grade III and atypical lobular hyperplasia according to classification in the histologic reports.

Dependent variables included ultrasound morphometric characteristics of LC. Independent variables include the histopathological results and age group of each patient. Confounding variables include the possibility of inter‐rater variability with the image analysis performed on the ultrasound images.

### Statistical analysis

A power calculation was done to meet the primary aim, considering the 5–20% incidence of LC^21^, ^22^ and the number of patients undergoing breast ultrasound procedures within the study setting.

A sample size of 100 was deemed appropriate to obtain a 95% confidence interval with a 5% margin of error, based on previous epidemiological studies conducted in the field of study.^21^, ^22^

Data analysis was performed in consultation with a biostatistician and a *P*‐value less than or equal to 0.05 was considered significant by means of Fisher's exact tests for all correlations that were analysed. Data analysis included frequencies and proportions to describe categorical variables.

For the ultrasound morphology descriptors, the chi‐square test was used to test for associations between the categorical variables.

For the comparison between morphology and histology descriptors, ultrasound characteristics were noted for each data set unit and correlated with the histopathology results. Fisher's exact test with a *P*‐value less or equal to 0.05 was considered as statistically significant results.

One‐way ANOVA was used to test for differences across age for the histological factors, namely Bonferroni correction and Bartlett's test for equal variances to assess whether there was homogeneity in variances.

The assumption was made that the image interpretation of the selected interpreters were accurate. All analysis was done using STATA 14 data analysis software.

## Results

The age range of the sample varied from 29 to 92 years with a mean age of 58.56 (±1.33) years. The frequency of the morphologic descriptions reported by the two interpreters is included in Table 1.

**Table 1:** Frequency of ultrasound morphology descriptions

Morphology description	Frequency (*n* = 100) (%)	Ratio
Shape		
Irregular	86	86.0
Round	1	1.0
Oval	13	13.0
Margin		
Circumscribed	5	5.0
Indistinct/poorly circumscribed	95	95.0
Microlobulated contour	49	49.0
Echogenicity		
Hypoechoic echogenicity	88	88.0
Hyperechoic	2	2.0
Complex/heterogenous echogenicity	10	10.0
Calcifications		
Absent calcifications	81	81.0
Present calcifications	19	19.0
Posterior acoustic features		
Posterior acoustic shadowing	93	93.0
Posterior acoustic enhancement	7	7.0
Lesion boundary		
Indistinct lesion boundary	52	52.0
Echogenic halo	30	30.0
Thin capsule boundary	18	18.0

**Figure 3:**
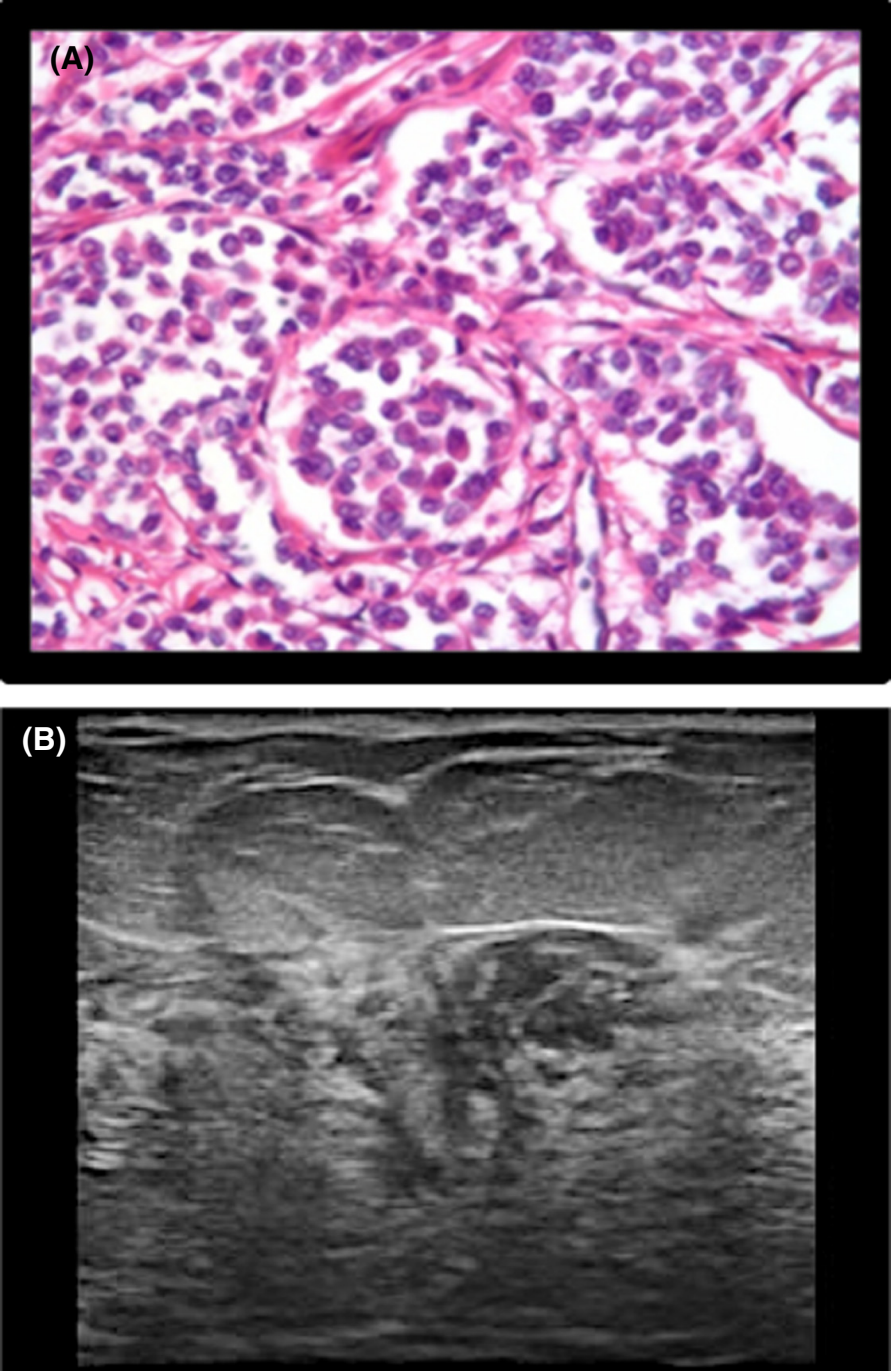
Histologic and sonographic appearance of an ‘ill‐defined mass’. (A) Histology: Classic type ILC. (B) Ultrasound image of ILC (ill‐defined mass).

The most frequently reported ultrasound morphology of LC cases included: an irregular (ill defined) ultrasound shape (86%) (see Fig. 3), hypoechoic echogenicity (88%), poorly circumscribed margin (95%), posterior acoustic enhancement (93%) as well as absent calcifications.

The histologic grade and description extracted from the histopathologic reports appear in Table 2.

**Table 2:** Frequency of histopathological findings

Histological grading:	Frequency (*n *=* *100) (%)	Histological description	Frequency (*n *=* *100) (%)
1: Lobular carcinoma Grade I	14	1: Epithelial hyperplasia	27
2: Lobular carcinoma Grade II	33	2: Stromal fibrosis	21
3: Lobular carcinoma Grade III	14	3: Signet ring appearance	9
4: ALH	39	4: Indian file/ribbons/single file	33
		5. Comedo necrosis/cystic dilated terminal lobular units	10

LC Grade II (33%) as well as atypical lobular hyperplasia/ LC in situ (39%) were groups with the highest frequency for histological grading as seen in Table 2. In addition the highest frequency of histological descriptions was epithelial hyperplasia (27%), stromal fibrosis (21%) as well as single file/single cell pattern/ ribbons (33%).

Morphologic characteristics that were the most common findings, were a hypoechoic mass as well as an irregular shape. This finding is supported by previous morphologic descriptions of LC.^23^, ^24^, ^25^ These two morphologic characteristics were analysed for association with histologic grade and description (Tables 3 and 4).

**Table 3:** Associations between irregular ultrasound shape, histological grading and description

Histological grading			Histological description		
Frequency (*n *=* *86)		*P*‐value	Frequency (*n *=* *86)		*P*‐value
ALH	31	<0.05	Indian file/ribbons/single file	31	<0.05
Lobular carcinoma Grade I	14	>0.05	Signet ring appearance	8	>0.05
Lobular carcinoma Grade II	31	<0.05	Epithelial hyperplasia/stromal fibrosis	39	<0.05
Lobular carcinoma Grade III	10	>0.05	Comedo necrosis/cystic dilated terminal lobular units	8	>0.05

Fisher's exact test: *P* ≤ 0.05 significant.

**Table 4:** Associations between hypoechoic ultrasound echogenicity, histological grading and description

Histological grading			Histological description		
Frequency (*n *=* *88)		*P*‐value	Frequency (*n *=* *88)		*P*‐value
ALH	33	<0.05	Indian file/ribbons/single file	31	<0.05
Lobular carcinoma Grade I	13	>0.05	Signet ring appearance	9	>0.05
Lobular carcinoma Grade II	30	<0.05	Epithelial hyperplasia/stromal fibrosis	29	<0.05
Lobular carcinoma Grade III	12	>0.05	Comedo necrosis/cystic dilated terminal lobular units	19	>0.05

**Figure 4:**
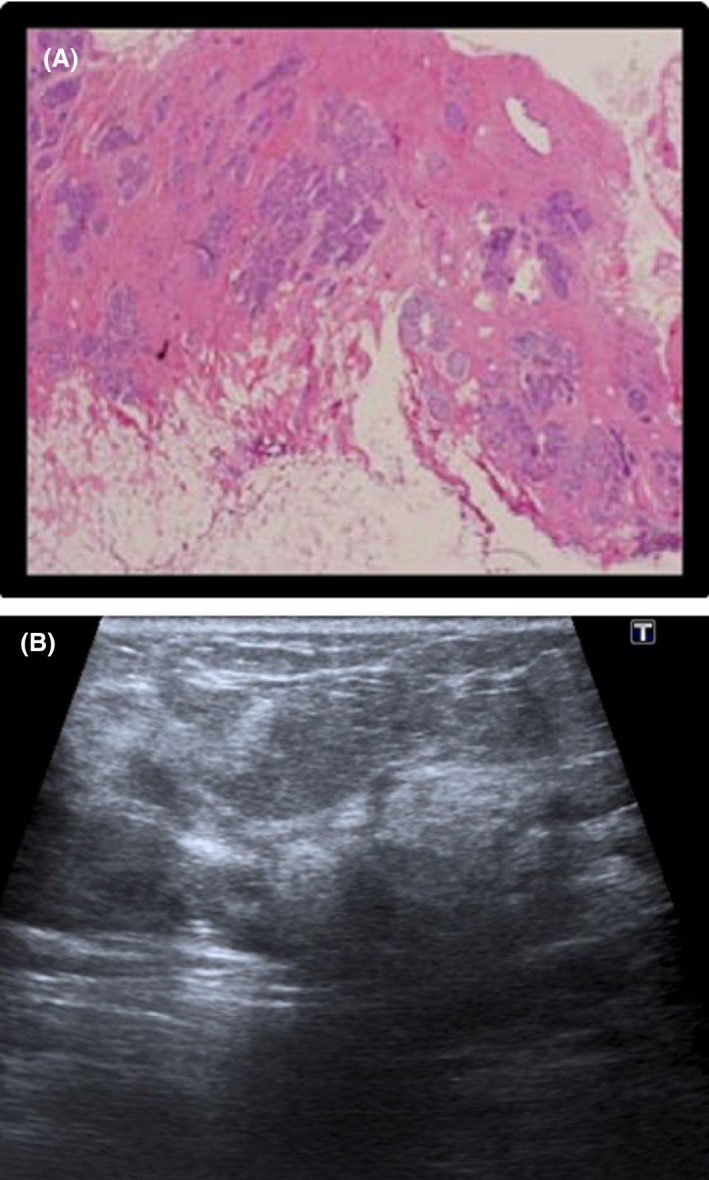
Histological and sonographic appearance of atypical lobular hyperplasia. (A) Histology specimen: Lobular Carcinoma Grade II. (B) Ultrasound image of LC Grade II: (Poorly circumscribed mass, ill‐defined borders).

**Figure 5:**
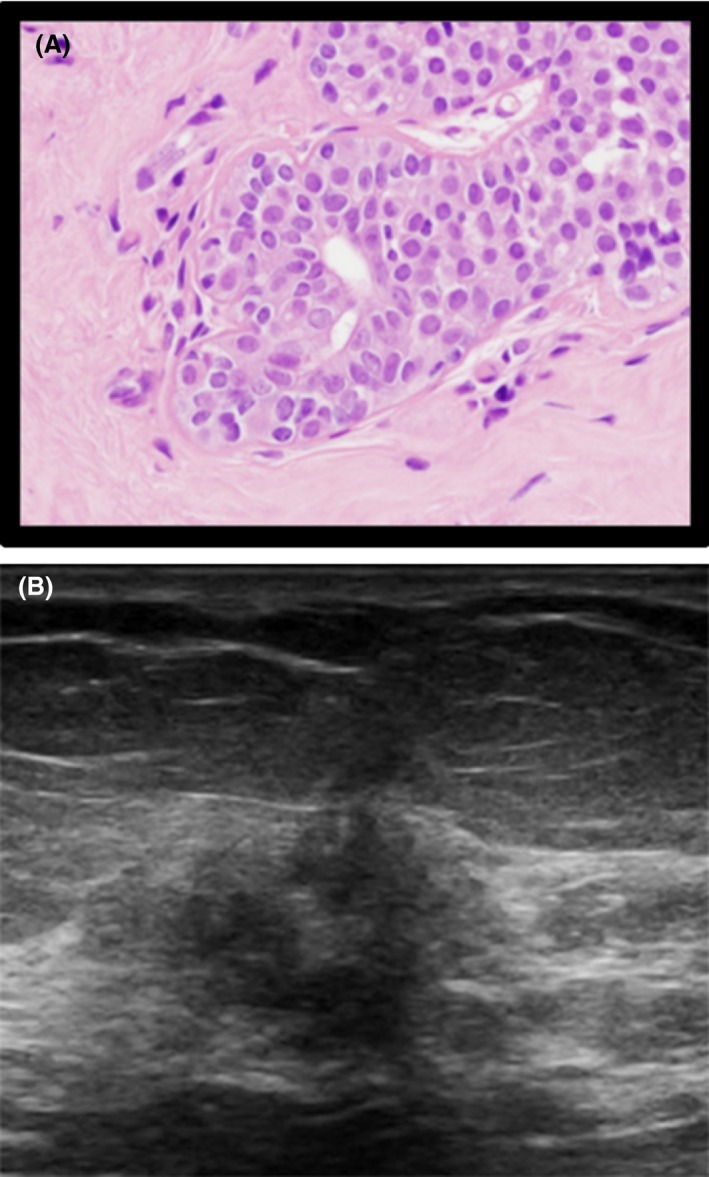
Histological and sonographic appearance of LC Grade II. (A) Histology specimen of ALH. (B) Ultrasound image of ALH (irregular, hypoechoic echogenicity).

An irregular ultrasound shape was significantly correlated (*P* ≤ 0.05) to atypical lobular hyperplasia (see Fig. 4) as well as to LC Grade II (see Fig. 5). Furthermore, there is a significant correlation between an irregular ultrasound shape (*P* ≤ 0.05) and the histological descriptions of single file/ribbons/single file patterns as well as epithelial hyperplasia or stromal fibrosis.

In terms of histologic grading, a significant correlation between hypoechoic echogenicity and atypical lobular hyperplasia as well as LC Grade II (*P* ≤ 0.05) was found. For histologic descriptions, there was a significant correlation between a hypoechoic ultrasound echogenicity, single file/ribbons/single file pattern as well as epithelial hyperplasia/stromal fibrosis.

## Discussion

There are multiple variations in the reported ultrasound morphologic properties of LC.

In the current study, morphologic properties included an irregular shape (86%) and hypoechoic ultrasound echogenicity (88%). These findings correlate with other studies, which described LC lesions as a hypoechoic,^20^ irregular mass^21^, ^22^ with ill‐defined margins.^1^, ^26^ In the current study, LC Grade II and atypical lobular hyperplasia were found to be the most common histologic grading. The incidence of LC Grade I was previously reported as 20%, with LC Grade II as 33% and LC Grade III as 14% which is nearly identical to the current study's findings.^6^

Histology descriptions unique to LC were single file type pattern which coincides with previous literature stating scattered columns of infiltrated malignant cells surrounding normal breast tissue in a diffuse pattern. However, this may not be seen consistently in all patients^10^, ^15^, ^16^

Weaknesses of the study included the use of a small sample size, as well as the interpreters not blinded to the study data; reducing its statistical significance.

The study is limited by its retrospective approach and acknowledges previous research regarding LC, in lieu of novel findings.

Larger sample sizes should be utilised in future research to improve the results related to inter interpreter agreement. With regards to the methodology; retrospective studies prove a possible selection bias with sample populations; future studies should attempt a prospective data collection approach.

## Conclusion

The use of both diagnostic and screening programmes in breast clinics should be implemented as standard protocol to improve patient predictor outcome. It should also be noted in practice, the importance of ultrasound as adjunct to mammography based on the current study's findings and should be practiced as part of a standard diagnostic screening programme tool.

In conclusion unique morphologic characteristics are related to histological findings of LC during ultrasound based diagnosis.

## Conflict of Interest

There is no financial support or relationships that may pose conflict of interest.
